# THE PORCHLIGHT PROJECT: COLLABORATING TO ENHANCE THE DEMENTIA CAPABILITY OF COMMUNITY-BASED VOLUNTEERS

**DOI:** 10.1093/geroni/igz038.1326

**Published:** 2019-11-08

**Authors:** Joseph E Gaugler, Gabriela Bustamante, Christina Rosebush, Jeri Schoonover, Roxanne Jenkins, Nicole Bauer, Lisa Beardsley, Laura Rowe

**Affiliations:** 1 University of Minnesota - School of Public Health, Division of Health Policy and Management, Minneapolis, Minnesota, United States; 2 University of Minnesota, Minneapolis, Minnesota, United States; 3 Lutheran Social Service of Minnesota, Saint Paul, Minnesota, United States

## Abstract

Public health efforts to address Alzheimer’ disease and related dementias (ADRD) are limited. Utilization of lay/peer intervention providers in the community to reach older persons and their families may offer a novel method to reach those in need. Such an approach may also serve as a fulcrum around which formal healthcare delivery and community-based LTSS are better integrated. This pragmatic trial, the Porchlight Project, aims to refine a multicomponent training approach for lay volunteers in Minnesota (i.e., Senior Companions) that enhances their capability to deliver dementia care and support to underserved older persons in need. This presentation will highlight the development and collaboration with Lutheran Social Services of Minnesota to refine and deliver a useful and feasible training program to enhance Senior Companions (n = 20) dementia care capability, as well as the potential and challenges of delivering a pragmatic trial of this type across the state of Minnesota.

